# Computational prediction of human fragile sites using genomic language models

**DOI:** 10.1093/bib/bbaf631.065

**Published:** 2025-12-12

**Authors:** Jomchai Chongthanakorn, Bingxin Lu

**Affiliations:** School of Computer Science and Electrical and Electronic Engineering, University of Surrey, Stag Hill, Guildford, GU2 7XH, United Kingdom; School of Biosciences, University of Surrey, Guildford, Stag Hill, GU2 7XH, United Kingdom; Surrey Institute for People-Centred Artificial Intelligence, University of Surrey, Guildford, Stag Hill, GU2 7XH, United Kingdom

## Abstract

Human fragile sites are genomic regions that are susceptible to DNA damage and breakage, especially under replication stress. They are strongly associated with genomic instability and play a critical role in diseases such as cancer and neurological disorders. Accurate detection of fragile sites can therefore advance our understanding of disease mechanisms and inform therapeutic strategies. However, only a few computational methods exist for predicting DNA fragility, and they typically rely on extensive feature engineering prior to inference. Genomic language models (gLMs), trained directly on raw DNA sequences, have recently emerged as powerful tools for diverse biological prediction tasks. Here, we explore the application of gLMs to predict fragile sites in the human reference genome.

We first constructed a balanced training dataset consisting of 3073 gene sequences located within manually curated fragile sites from the HumanCFS database and an equal number of negative sequences sampled from random genomic regions. Using a 70–10-20 train-validation-test split of this dataset, we evaluated the performance of three gLMs (Caduceus, HyenaDNA, and Nucleotide Transformer), together with a convolutional neural network, to predict fragile sites.

Our results show that fragile sites exhibit distinctive nucleotide-level patterns that can be effectively learned by deep neural networks, particularly those modelling long-range interactions. Beyond predictive accuracy and efficiency, interpretability analyses and embedding visualisations indicate that these models capture biologically meaningful patterns. Future work will expand this framework with larger datasets and extend its scope to predicting rearrangement hotspots and genome-wide DNA fragility.

